# Role of sleep duration and obesity-related health behaviors in young children

**DOI:** 10.1016/j.pmedr.2020.101199

**Published:** 2020-09-04

**Authors:** Julie Gazmararian, Jonathan Smith

**Affiliations:** Department of Epidemiology, Rollins School of Public Health, Emory University, Atlanta, GA, United States

**Keywords:** PA, Physical Activity, ECE, Early Care and Education centers, Sleep, Physical activity, Early childcare, Obesity

## Abstract

Adverse health outcomes and obesity are especially high among children with sleep deficiencies. Such health consequences may differentially affect children 2–5 years of age, during which time children undergo drastic shifts in sleep patterns and neurological development. To date, little epidemiologic research has examined the link between sleep and obesity-related health behaviors among this age group. We investigated the relationship between sleep and eating/physical activity behaviors among children aged 2–5 years old. In fall 2018, parents and teachers of 1,169 children attending 34 Early Childcare Education (ECE) centers in Georgia completed surveys on sleep patterns and obesity-related health and eating behaviors. In this analysis, sleep was not associated with various child eating behaviors either at home or in the classroom, however, shorter sleep duration was found to be associated with lower physical activity during the school day (p < 0.01). Furthermore, when stratifying by gender, girls sleeping <10 h were less likely to eat more than one vegetable at home than those sleeping ≥10 h (p = 0.04). This preliminary evidence is a starting point, from which researchers may continue elucidating the drivers of obesity in this understudied population.

## 1 Introduction

The links between pediatric obesity and serious immediate and long-term cardiovascular, metabolic, and other health consequences during childhood are well-established (Reilly et al., 2003, Reilly, 2005). Sleep plays a key role in childhood obesity, such that insufficient sleep duration is strongly associated with a higher risk of pediatric obesity (Kobayashi et al., 2012, Patel and Hu, 2008, Chen et al., 2008). In younger children, there is a strong dose-response relationship between insufficient sleep duration and increased obesity (Chen et al., 2008). Such findings highlight the need to identify early life factors contributing to pediatric obesity, such as physical activity (PA), eating behaviors, and socioeconomic status (Rogers et al., 2015). Several research studies have investigated the relationship between sleep and dietary intake in children; a recent systematic review and meta-analysis found strong associations between shorter sleep duration and unhealthy eating habits in older children (Córdova et al., 2018). However, these conclusions were overwhelmingly drawn from children above five years of age and adolescents. Unfortunately, little epidemiologic research has examined the relationship between sleep duration and obesity-related health behaviors among preschool-aged children (2–5 years old). It is during this period of life when children undergo drastic changes in physical, social, psychological, and emotional growth. In this context, we conducted a survey on sleep behavior and obesity-related eating and health behaviors among children 2–5 years of age attending Early Childhood and Education (ECE) facilities in greater Atlanta, Georgia between 2 and 5 years of age. Surveys on eating and physical activity behaviors were separately distributed to guardians and ECE center teachers. The primary objective was to evaluate nocturnal sleep duration and differences in these obesity-related behaviors (diet and PA).

### 1.1 Materials and methods

#### 1.1.1 Setting

Participants were recruited from 34 ECE centers in Georgia, representing a range of enrollment size, geographic areas, and demographics.

#### 1.1.2 Data sources

In Fall 2018, two surveys were distributed to the targeted ECE centers assessing the child’s nutrition and PA behaviors. One survey was completed by family members who were picking up or dropping off their child(ren). The second survey was completed by teachers for the children whose family members had completed surveys.

#### 1.1.3 Study measures

The family member survey contained closed-ended prompts focusing on the child’s and family member’s health and eating behaviors, as well as the child’s gender and age. Questions assessed the child’s daily dietary intake (multiple fruit, multiple vegetables, water, and sugary drinks/‘soda’), physical activity, bedtime, and waketime. Response options for dietary intake prompts were: “Never,” “Rarely,” “Some Days,” “Most Days,” and “Every Day.” For PA, parents indicated the number of days per week the child was physically active (range 0–7). Bedtimes and wake times were provided in 30-minute increments. Teacher surveys contained a subset of these questions applicable to an ECE setting, specifically fruit, vegetable, water intake, and PA. PA in the teacher survey was recorded using the same five response options for dietary intake. Sugary drinks/soda behavior was not assessed in the teacher surveys since sugary drinks and sodas are not provided at ECE centers. Nocturnal sleep was not assessed in the teacher survey since it is not applicable in an ECE setting.

#### 1.1.4 Analyses

ECE center-level data on race, reduced-lunch eligibility, and number of students and teachers were summarized.

In our primary analyses, we followed established guidelines for sleep duration of children 3–5 years old (“<10 h” or “≥10 h”) (Sherrill et al., 1998). We further combined the “rarely” and “never” categories for the dietary questions due to the low frequency of responses. Parent-reported child PA data were reassigned in the following manner: values of 0–1 as “Rarely/Never”, 2–3 as “Some Days,” 4–5 as “Most Days”, and 6–7 as “Every Day.” The child’s nocturnal sleep duration was calculated from the difference in sleep and wake times. The nonparametric Wilcoxon Rank Sum Test was used to evaluate if there was a statistically significant difference between the health behaviors of children who slept less than 10 h and the health behaviors of children who slept 10 h or more.

The association between sleep duration and obesity-related eating and PA behaviors was further assessed using additional analytic approaches. Since a disproportionate amount of the sleep data (30%) indicated exactly 10 h of sleep, we also categorized sleep duration into three categories, “<10 h,” “10–11 h,” and “>11 h.” In this scenario, we used the nonparametric Kruskal-Wallis test to identify statistically significant differences between activities and sleep categories. Moreover, for both approaches to categorizing sleep duration (dichotomous and trichotomous), we further evaluated associations between sleep and the full distribution of nutrition and PA responses (1–5 for nutrition and classroom PA, or 0–7 for home PA). We also considered sleep duration as a continuous variable and individually evaluated the activity scores as ordinal predictors in a simple linear regression model. Since results were similar for the different categories of sleep, we only present results using the dichotomous categories. Anonymized data and analytic code to recreate associative measures for all primary and secondary analyses are available upon request. Lastly, each one of these analyses were further stratified by child age and gender.

### 1.2 Results

A total of 1,265 children had parents complete the survey, of which 1,195 (95%) had sleep data available. An additional 26 (2%) children were excluded as they did not have teacher reported child behavior in the classroom, yielding a final analytic sample of 1,169 (92%) children.

Among parents responding to questions on children’s age and sex, the median age was 4 years (Interquartile Range [IQR]: 3–5 and 47% of children were female (Table 1). Almost all (98%) students at participating ECE facilities qualified for free or reduced lunches under the income-based Georgia Department of Education policy. (Chasens et al., 2011) The median number of students per teacher across all facilities was 7.1 (IQR: 6.1, 9.6).

**Table 1:** Demographic characteristics of the child-level analytic sample and ECE centers completing survey.

Characteristic	Child-level data (n = 1,169)*	ECE Centers (n = 34)
Age (Years), median (IQR)*	4 (3–5)	4 (3–5)
Female*	47%	50%
Race		
Black	–	56%
White	–	32%
Hispanic	–	11%
Other†	–	1%
Free/reduced lunch eligibility	–	98%
Students per teacher, median (IQR)	–	7.1 (6.1, 9.6)

* Not all parents responded to demographic prompts; n = 822 (70%) and n = 807 (69%) for age and gender, respectively.

† “Other” category includes American Indian, Pacific Islander, Asian, and Multiple Races.

**Fig. 1:**
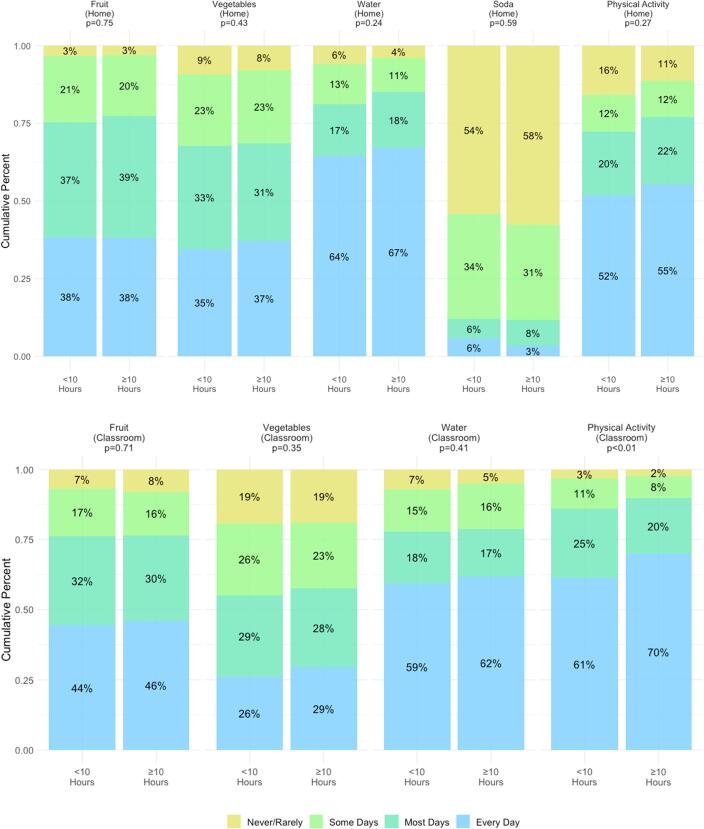
Obesity-related health behaviors at home and in the classroom, by sleep category. Top panel indicates at-home behaviors as reported by the parent survey, bottom panel indicates in-classroom sleep behaviors as reported by the teacher survey. The Wilcoxon sum-rank test was used to calculate p-values.

Fig. 1 shows the results from the primary analysis of dichotomized data among the entire cohort. In all analytical approaches of sleep and health behaviors data, PA in the classroom was the only activity significantly associated with sleep duration (p < 0.01 for all analyses). The relationship indicates that reduced sleep duration is associated with lower levels of PA during the school day. In all analyses of the total cohort, there was no significant difference in multiple fruit, multiple vegetable, water, and soda intake between children who slept less than 10 h and children who slept 10 h or more.

As not all children had age and gender data, stratified analysis reduced our stratified analytic cohort (n = 822 and 807, respectively). Before stratification, we first re-examined the above relationships among these smaller samples and found identical results as the full analytic sample. Further stratifying by age did not yield additional associations between sleep and health behaviors. When stratifying by gender, girls sleeping < 10 h were less likely to eat more than one vegetable at home than those sleeping ≥ 10 h (p = 0.04). This association held when analyzing both the original responses and the recoded (categorized) responses as described in methods.

### 1.3 Discussion

In this cross-sectional analysis we examined whether sleep duration was associated with obesity-related health behaviors at home and in the classroom. While we found that sleep duration was not associated with most diet and physical activity behaviors in either setting, the notable exception was physical activity in the classroom. Throughout all combined and stratified analyses, children with shorter sleep durations were more likely to report less physical activity in the classroom. Stratifying by gender also revealed that longer sleep duration was associated with increased vegetable intake among girls.

It is well established that physical activity is inversely related to adiposity and obesity among children and preschoolers (Shi et al., 2010, Parsons et al., 2018). While our findings concur with the long-established evidence identifying a bidirectional relationship between sleep duration and physical activity among adults and older children in other settings (Sherrill et al., 1998, Chasens et al., 2011, Shi et al., 2010), there are few studies investigating this specific relationship among preschool-aged children specifically. are more limited. However, a recent study during the same timeframe and in comparable settings found a similar association between nocturnal sleep (in hours) and total light, moderate, and vigorous physical activity (p = 0.01) (Parsons et al., 2018).

While increasing evidence demonstrates a clear connection between insufficient sleep and obesity across all age groups, less work has examined specific mechanisms that may explain this connection among preschool-aged children. A 2012 study by Clifford *et al* examined the association between sleep duration and caloric intake and found longer sleep duration was associated with lower caloric intake (Clifford et al., 2012). Importantly, this study only recruited obese preschoolers already enrolled in a weight management program. Another study implemented an experimental protocol to examine changes in sleep patterns and dietary intake, and found that acute sleep restriction was associated with higher caloric intake among preschoolers (Mullins et al., 2017). Although the findings were statistically significant, the total sample population was only 10 children. However, these findings do concur with another study which found shorter sleep duration was associated with greater fat and decreased carbohydrate intake among preschool aged children (Petrov et al., 2017).

The purpose of this analysis was to contribute to the preliminary body of evidence investigating specific obesity-related health behaviors and sleep duration and is subject to several key limitations. Primarily, our measures of dietary intake and physical activity were broad; the use of fruit, vegetable, and soda intake were crude proxies for healthy eating behaviors. Moreover, we only collected data on simple daily frequency. There may be considerable heterogeneity in the number of hours of physical exercise or caloric intake among children each day. As a result, these findings are intended to inform future research and caution should be exercised when interpreting results for specific decision making. Additional research is necessary to more closely examine the underlying mechanisms of sleep and obesity among preschoolers; and should include data on family physical activity and bedtime habits, family risk factors and co-morbidity, birthweight and gestational age. Moreover, our data were self-reported, and studies obtaining sleep and physical activity using more objective data sources may improve our understanding of this relationship. Lastly, our data were among lower-income families in Georgia and may not be generalizable to external populations; more representative samples are needed.

Early childhood is a complex period of physical, emotional, social, and cognitive growth. Children’s sleep patterns undergo dramatic shifts from age two to five. By five years old, sleep patterns more closely resemble adults than toddlers. This analysis underscores the association between sleep and obesity-related health behavior in a low-income population at risk for higher rates of obesity.

## CRediT authorship contribution statement

**Julie Gazmararian:** Conceptualization, Methodology, Writing - review & editing. **Jonathan Smith:** Writing - original draft.

## Declaration of Competing Interest

The authors declare that they have no known competing financial interests or personal relationships that could have appeared to influence the work reported in this paper.
